# The Classroom Communication Resource (CCR) intervention to change peer’s attitudes towards children who stutter (CWS): study protocol for a randomised controlled trial

**DOI:** 10.1186/s13063-017-2365-x

**Published:** 2018-01-17

**Authors:** Rizwana Mallick, Harsha Kathard, Lehana Thabane, Mershen Pillay

**Affiliations:** 1 University of Cape Town, Cape Town, WC South Africa; 2 McMaster University, Toronto, Canada; 3 University of KwaZulu Natal, Durban, South Africa; 4 University of Cape Town, Private Bag X3, Rondebosch, 7701 South Africa

**Keywords:** Stuttering, classroom-based, interventions

## Abstract

**Background:**

Children who stutter (CWS) are at a high-risk of being teased and bullied in primary school because of negative peer attitudes and perceptions towards stuttering. There is little evidence to determine if classroom-based interventions are effective in changing peer attitudes towards stuttering. The primary objective is to determine the effect of the Classroom Communication Resource (CCR) intervention versus usual practice, measured using the Stuttering Resource Outcomes Measure (SROM) 6-months post-intervention among grade 7 students. The secondary objective is to investigate attitude changes towards stuttering among grade participants on the SROM subscales.

**Methods:**

A cluster randomised controlled trial (RCT) will be conducted with schools as the unit of randomization. Schools will be stratified into quintile groups, and then randomized to receive the CCR intervention or usual practice. Quintile stratification will be conducted in accordance to the Western Cape Department of Education classification of schools according to geographical location, fee per school and allocation of resources and funding. Participants will include primary schools in the lower (second and third) and higher (fourth and fifth) quintiles and children aged 11 years or older in grade 7 will be included. The study will consist of the CCR intervention program or usual practice as a no-CCR control. The CCR is a classroom-based, teacher led intervention tool including a story, role-play and discussion. The grade 7 teachers allocated to the CCR intervention, will be trained and will administer the intervention. The analysis will follow intention-to-treat (ITT) principle and generalized estimating equations (GEE) to compare groups on the global SROM and its subscales to account for possible clustering within schools. The subgroup hypothesis will be tested by adding an interaction term of quintile group x intervention.

**Discussion:**

This study is designed to assess whether the CCR intervention versus usual practice in schools will lead to positive shift in attitudes about stuttering at 6-months post-intervention among grade 7 participants.

**Trial registration:**

The trial number is NCT03111524. It was registered with clinical trials.gov Protocol registration and results system (PRS) retrospectively on 9 March 2017.

**Electronic supplementary material:**

The online version of this article (10.1186/s13063-017-2365-x) contains supplementary material, which is available to authorized users.

## Background

### Children who stutter

Children who stutter (CWS) are placed at risk for being teased and bullied in primary school due to negative peer attitudes and perceptions [1–4]. Negative attitudes and interactions result in CWS being viewed as different or disabled, leading to social rejection [3]. Social rejection may lead to long term negative consequences such as reduced academic and social interactions, depression, and negative self-perceptions [5, 6] which are harmful if not urgently addressed. These consequences are particularly prevalent in the adolescent population due to stress and rapid changes of emotion at this age [3].

### Attitudes and attitude change

Attitudes and perceptions overlap [7] which is important to consider as negative peer attitudes may lead to negative perceptions towards CWS [1–4]. While the relationships between attitudes, attitude change and behaviour change are complex and multifaceted [8], this study focusses on attitude as the precursor for behaviour change [9] but does not focus on behaviour change. The underpinning of attitudes for this study considers how literature characterises it. Attitudes are described as an individual’s evaluation of issues, objects and other individuals [8]. As such, the evaluation of another person or object can be positive or negative [10]. It is additionally reported that attitude formation is known to continuously change over time, [11] as it is learnt and shaped [7].

### Stuttering intervention

The International Classification and Functioning of Disability (ICF) framework [12] considers holistic management of the CWS. It advocates for classroom-based interventions to reduce teasing and bullying [12–14, 15] because children spend a large amount of time with their teachers [16]. Classroom-based interventions therefore aims to advocate for CWS and to empower teachers as communication partners of Speech-Language Therapists (SLTs) and CWS as guided by population-based stuttering interventions.

International public education is another population-based campaign that was studied. It addresses stuttering-related stigma [9] through reducing the debilitating nature of stuttering and improving social environments and reactions [17]. These publicised campaigns have, however, not documented effectiveness [9]. Despite these findings,, the potential for classroom-based interventions to change attitudes towards stuttering are emerging [18–22] and supported by the following studies: the Public Opinion Survey of Human Attributes- Stuttering (POSHA-S) internationally and in South Africa and the Teasing and Bullying: Unacceptable Behaviour (TAB). The international POSHA-S study showed that negative attitudes are in fact prevalent in school-aged children [23]. A follow-up study conducted in South Africa, using the POSHA-S, showed that teachers were also requesting assistance with managing negative attitudes towards stuttering [24]. Another tool used to address peer negative attitudes towards stuttering was the TAB which included teacher administered activities and yielded positive results pre- and post- intervention [14, 15, 25]. The TAB was, however, not suitable for South African classrooms due to time and technology constraints as well as contextual, cultural and linguistic differences.

This led to the development of the South African specific intervention, the CCR intervention. It was developed and has been refined since 2009 as part of a series of the University of Cape Town (UCT) projects. The CCR intervention yielded positive results at 1 month post-intervention within the lower and higher quintile population respectively [18, 26] and more so at 6 months’ post-intervention [19]. The feasibility of a future Randomised Controlled Trial (RCT) study additionally reported potential effectiveness of the CCR intervention at 1 and 6 months’ post-intervention as well as procedural aspects [19]. The findings were however inconclusive as it called for a more rigorous design method [19]. It was also reported that a RCT was feasible despite concerns regarding the retention of participants as stringent methods could be put in place [19]. A RCT was thus recommended as the next stage in these projects [19].

The CCR intervention addresses pro-social behaviours and skills, including but not limited to the promotion of positive behaviour change, peer support and resilience through intervention [16] in the areas of Positive Social Distance (PSD), Verbal Interaction (VI) and Social Pressure (SP) in the CCR intervention and Stuttering Resource Outcomes Measure (SROM). The areas of PSD, VI and SP are additionally measured using the SROM. PSD represents the overall ease, acceptance of and comfort a child feels when around CWS [14, 15] e.g. ‘I would let a child who stutters hang out with us’. VI evaluates peer’s negative thoughts, emotions and feelings, e.g. frustration experienced towards a CWS [14, 15]. SP evaluates general thoughts regarding CWS through examining social pressure and subjective norms [13]. An example is ‘I would be ashamed to be seen with a child who stutters’. The promotion of these pro-social behaviours and skills may facilitate the prevention of anxiety and depression [16] especially as CWS are placed at a high-risk of being teased and bullied due to their stutter [1–4].

## Objectives

### Primary objective

This study aims to determine the treatment effect of the CCR intervention versus usual practice (i.e. no CCR) using the SROM global score at six months’ post-intervention among grade 7 participants in different schools.

It is hypothesised that the CCR intervention will result in a positive shift in the treatment effect in the intervention groups at 6 months’ post-intervention. The intention of the CCR intervention is to improve the participants’ attitudes around stuttering, teasing and bullying while encouraging the acceptance of the diversity amongst peers.

### Secondary objectives

The secondary objective is to determine the treatment effect on attitudes towards stuttering among Grade 7 participants based on the SROM subscales of PSD, SP and VI.

It is hypothesized that there may be an improvement in each subscale but it is unknown which will show a greater improvement. These subscales will be compared to the control group treatment effect. This study will also evaluate the areas of interaction between peers that exist and may be self-perceived.

The subgroup analysis objective is also to determine the primary objective between and across the lower and higher quintile school clusters. Previous studies have shown that the lower quintile schools are more negative than the higher quintile schools initially [19]. It is hypothesized that both quintiles in the intervention groups will yield positive shifts in treatment effects when compared to the control group while it is unknown which quintile will be most positive, or where the greatest shifts will occur.

## Methods

### Trial design

This study will make use of a stratified cluster randomised controlled trial with the schools as the unit of randomisation. Using a 1:1 allocation ratio, schools will be stratified into two quintile groups (lower versus higher). The quintiles will be randomised, to receive the CCR intervention or usual practice, to eliminate selection bias and to control for any extraneous variables. This will also allow each lower and higher quintile school an equal opportunity to be included (See Additional file 1: Figure S2).

### Overview of the South African and study context

#### Study setting and participants

The South African educational study context is influenced by its socio-political history. Post-apartheid schools remain unequal, particularly in relation to resources. In an attempt to address this inequality, a system based on the National Norms and Standards for school funding (NNSSF) policy was developed to classify schools in relation to resources, [27, 28] fee per schools, funding and geographical location. For example, lower quintile schools one, two and three are classified as no fee-paying schools [29, 30] while higher quintile schools four and five are fee-paying schools that are better resourced [29, 30]. This study therefore aims to compare the treatment effect in the lower and higher quintile schools, explored as a subgroup in this study, to ensure that the schools are representative of the country’s educational context.

Participants from public schools, in lower and higher quintiles, within the Western Cape metro urban area, in South Africa are therefore included. Schools with an onsite SLT will not be included as they may have already addressed teasing and bullying related to CWS in the school context.

### Eligibility criteria

Eligible participants for the primary objective of this study include grade 7 mixed- gender participants aged 11 years and older attending public schools within the Cape Metro urban area across the lower (two and three) and higher (four and five) quintiles where the Language of learning and teaching (LoLT) is English. Participants will not be compensated financially for their time. All schools who participate in this study, will be provided with a copy of the CCR intervention. The exclusion criteria for this study will include private primary schools in the Cape Metro urban area, and schools that do not have mixed-gender participants.

### Intervention

The CCR intervention consists of a social story, role-play, and a semi-structured teacher-led discussion. It will be administered by the class teacher and will require active participation of learners. The teacher will be required to read the story to the class. Thereafter, she will select students in her class to perform the role-play. The role-play contains the same story plot of the story that she will have read to the class. This was purposely done in this manner in order to physically put the study’s participants in the characters “shoes”. The teacher-led discussion will include guidelines on which topics should be covered, however, teachers may also explore these topics in greater depth if they would like to.

The CCR intervention will be administered to the intervention groups only. While the CCR intervention is aimed to be largely self-sufficient and user friendly, it is a supported guide and thus teacher training will be required. Training will be required specifically around the discussion activity as teachers may require assistance with targeting the issues around diversity, difference, race and culture in the area of communication and stuttering. Queries and concerns will be addressed as part of the training. Teachers in the intervention groups will be encouraged to answer questions that arise from participants around the CCR intervention, discuss the questions and make notes in a logbook for the researcher.

The CCR intervention will only be administered once, as it is a single-dose intervention tool. The researcher will observe how teachers administer the CCR intervention and take notes during this time. The teacher will be left to administer the CCR intervention as she was trained and without interference from the researcher at this stage. The teacher may provide voluntary feedback at that stage. The teacher will also be given the opportunity to discuss their experiences with the researcher after the 6 months’ post-intervention data is collected.

### Control

The participants that are randomised to the control group, will not receive any intervention. Teachers’ in the control group will be encouraged to continue with their daily activities as normal, without drawing attention to stuttering discussions. However, if questions arise from participants, they are to answer the questions and make notes of any discussion that occurs related to stuttering, teasing and bullying. Control groups will receive a copy of the CCR intervention and will be provided with training once the study is complete.

### Outcome measure

#### Sampling and enrolment

Continuous sampling is impractical as this study is concerned with participants at 6 months’ post-intervention. Thus once-off randomised sampling will occur to track treatment effect from pre-intervention to 6 months post intervention using the same participants.

#### Primary outcomes

The primary outcome endpoint of this study will be to observe a positive shift, in magnitude and direction, of the treatment effect at 6 months post intervention from pre-intervention in the intervention groups only. This will be calculated by using the SROM to compare the ratings of peer attitudes at pre-intervention and at 6 months post intervention. This will also be explored in terms of the subscales of the SROM as well as the comparison of the lower quintile- to the higher-quintile schools. The SROM will be able to evaluate the primary objective as well as the secondary objectives related to the treatment effect in subscales and the subgroup analysis between quintiles.

The SROM was developed as a South African specific outcomes measure as a modification of the Peer Attitude Towards Children who Stutter (PATCS). The PATCS met the suggested criterion reliability [14, 15] and so did the SROM [18]. Evidence of the validity and reliability of the SROM was conducted and reported through a number of UCT thesis manuscripts that are available online [18, 20–22]. After a research panel of SLTs selected questionnaire items [20], cognitive debriefing sessions were held with grade-7 participants [20, 21] and the SROM was tested and finalised [22]. The reliability and validity of the SROM was evaluated where the following was noted: construct validity yielded a shift in the intervention group only after the intervention was administered; the internal consistency reliability score was 0.94; and the test-retest reliability was found to be 0.84.

The SROM consists of a 5-point Likert Scale including 20 items and four unrelated practice items. It includes three psychometrically approved constructs, as previously discussed – PSD, SP and VI – that represent attitudes [4]. PSD refers to the comfort, overall acceptance and ease that a child feels around a CWS [14, 15]. An example of an item found in the PSD construct is ‘I would let a child who stutters hang out with us’. An example of a SP item is ‘I would be ashamed to be seen with a child who stutters’. As illustrated by the example, SP refers to the general thoughts about a CWS through evaluating subjective norms and social pressure [4]. VI, refers to negative feelings, thoughts and emotions that are experienced towards a CWS. This could include frustration [4]. For example, a question in this subscale includes ‘listening to a child who stutters would annoy me’.

### Participant timeline

**Fig. 1:**
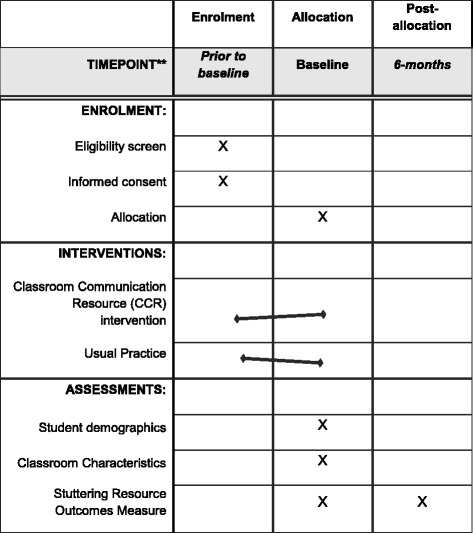
Timeline for trial activities, interventions and assessments

The data collection procedure will include enrolment, interventions after baseline (pre-intervention) and assessments at pre-intervention and 6 months post intervention (see Additional file 2: Standard Protocol Items: Recommendations for Interventional Trials (SPIRIT) Checklist, the SPIRIT Figure (Fig. 1) and Additional file 1: Figures S2, S3 and Table S1).

### Enrolment

Following ethical approval and permission being obtained, schools will be contacted to be recruited to participate. Once all schools agree to participate, randomisation will occur.

### Interventions

All participants will view a video of a CWS and stuttering will be defined in order to ensure that all participants are provided with a uniform definition of a CWS in terms of how it looks and sounds. A 1-h training session (administration guidelines, purpose and aims, discussion ideas, addressing questions) will be held with the teachers in the intervention groups only, once they reviewed the CCR intervention. Teachers will be given a 2-week period to review the CCR intervention again before they administer it. Control groups will not receive the CCR intervention during this phase.

### Assessments

The SROM will be administered at pre-intervention and again at 6 months post intervention. The limitation of using the SROM repeatedly over the 6-month period is acknowledged. In the absence of an equivalent validated measure, the SROM will be used as there will be a time lapse between administrations reducing potential re-intervention bias. Once the data is collected, teachers from the control groups will be provided with the CCR intervention and will be offered training.

### Sample size with power analysis

The sample size calculation was based on previous CCR intervention studies [18, 19, 26]. With the proposed sample size of *n* = 350 students (*k* = 10 schools) in each of the two groups (i.e. assuming a 1:1 allocation ratio), the study will have the power of 80% to yield a statistically significant result using a generalised estimating equations (GEE) model (assuming an intention-to-treat (ITT) principle for the analysis) of the difference between mean in SROM global scores at 6-month adjusting for baseline (pre-intervention) SROM global score at alpha = 0.05. This computation is based on a pilot study [19] which assumes that SROM global scores are normally distributed, the mean difference is 5.25 (corresponding to mean of 77.91 (for the intervention group) versus 72.66 (for the control group)) and the common within-group standard deviation is 11.90 and an ICC (intra-school correlation coefficient) of 72.70.

### Recruitment

To achieve adequate enrolment of participants, school recruitment will be conducted which is scheduled to commence on 19 January 2017. Thereafter, the returned consent and assent forms will determine whether the target sample size was achieved. If it has, then no more schools will be recruited.

### Assignment of interventions

#### Allocation: sequence generation, allocation concealment mechanism and implementation

The statistician will generate the computerised allocation sequence. The random allocation ratio will be 1:1 while the randomisation will be stratified into 2:1 where the lower quintile has a higher number of assignments of participants per school when compared to the higher-quintile schools. The written allocation of the assignment of participants will be sealed in an envelope which contains identification numbers. These identification numbers will be distributed across the lower- and higher-quintile schools. A sufficient number of schools will be included to meet the targeted sample size. The researcher will open the envelope to determine the allocation of schools. The stratification of the sample will occur in the following order; mixed-gender schools, schools within quintiles 2 and 3 and 4 and 5 and finally according to the eligibility characteristics and restrictions.

### Blinding

The principal investigator will be fully blinded to the study. The primary researcher will complete the following: obtain permission from the relevant individuals; recruit participants; recruit research assistants; assist research assistants with training of intervention group teachers to use CCR intervention and; observe the administration of the CCR intervention only along with research assistants. A team of research assistants will be utilised to assist with randomisation and blinding of the primary researcher regarding the administration and data capturing of the SROM.

### Data collection, management and statistical analysis

#### Data collection

As mentioned, the SROM will be administered pre intervention and will be administered 6 months post intervention to all participants. Participation retention will be promoted through rigorous planning of arranging data collection at times and dates most convenient for the schools.

#### Data management

The Consolidated Standards of Reporting Trials (CONSORT) Statement will be used when reporting on the trial. Imputation will be included related to the missing data according the cause for missing data such as absenteeism. Raw data will be captured on a Microsoft Excel spreadsheet using allocated coded numbers that will be used during data collection. Control and intervention groups will be captured on two separate spreadsheets and within each group, each quintile will be included. Answers for each question will be included on the spreadsheet using a number between 1 and 5 (strongly disagree = 1, disagree = 2, not sure = 3, agree = 4 and strongly agree = 5). Negative items will be reversed scored (e.g. strongly disagree will be 5). Total SROM mean scores, i.e. global scores, will be calculated at this point. Data will be cross-checked between the research assistants and then rechecked independently by another research assistant to minimise errors. Discrepancies in the data capturing will be reviewed and rechecked and corrections will be made where applicable. According to the Guidelines for Good Practice in the Conduct of Clinical Trials with Human Participants in South Africa, it is recommended that the data be kept for 15 years after the formal discontinuation of the trial [31]. The principal investigator will be responsible for securely storing the data as well as discarding the data.

#### Statistical methods

The analysis will follow the intention-to-treat (ITT) principle and will be reported according to CONSORT guidelines [32]. The GEE will be used to compare the groups on global SROM and subscales to address the primary objective. Assuming an exchangeable correlation structure within a school, GEE will allow for possible clustering within a school to be accounted for. The unit of analysis will be the grade-7 student. The results will be reported as estimate of the difference between groups, 95% confidence interval and associated *p* value. All *p* values will be reported to three decimal places with those less than 0.001 reported as *p* < 0.001. The criterion for statistical significance will be set at alpha = 0.05. The subgroup hypothesis will be tested by adding an interaction term of the quintile group (lower versus higher) × intervention (CCR versus usual practice) in the model. Similarly, this analysis will be used to address the secondary objective by analysing the PSD, VI and SP constructs on the SROM. The subgroup analysis will use this method of analysis to address the subgroup analysis between and across the quintiles. See Additional file 1: Table S1 for a summary table of the objectives, outcomes, hypotheses and methods of analysis.

#### Subgroup analyses

Subgroup analyses between quintiles and cluster analysis between the sample has been explored in a previous feasibility study [19]. It was found that schools behaved as clusters and thus it was appropriate to administer and evaluate participants within clusters [19], supporting a group-based approach. There are no findings between quintiles using the CCR intervention in such a large-scale trial and thus this study aims to include this aspect of analysis.

#### Nested studies

A few challenges were highlighted in the previous study such as poor retention of participants [19]. It is unknown what other challenges may arise. It is for this reason that the researcher, research assistants and teachers will be required to have logbooks in which detailed accounts and experiences are documented.

### Monitoring

#### Data monitoring and auditing

The data will be captured, audited, monitored and secured by a team of research assistants along with a statistician who will form the Data Monitoring Committee. The research assistants and statistician have no competing interests.

## Discussion

### Harms

The teacher will be consulted prior to data collection to identify whether there is a CWS in the classroom. The CWS may choose to not participate or to not have the intervention run in their classroom. Teachers will also be asked to note any concerns. Any participants requiring counselling will be referred to a psychologist by the researcher. However, in all the previous studies, no concerns or need for counselling have been identified or required [18, 19]. In fact, the studies showed minimal improvements in the ratings of attitudes at 1 month post intervention while more prominent results were observed at 6 months post intervention [18, 19]. Other benefits include access and training to the CCR intervention for all schools and teachers. The benefits, therefore, outweigh any potential risks that may be experienced. The data will be collected at schools where participants are comfortable, and the use of logbooks will be vital in informing if any harms are noted.

### Ancillary and post-trial care

Post-trial care will include the provision of the CCR intervention and training to control group teachers. No other harms are anticipated, as mentioned previously.

### Dissemination policy

The primary researcher will provide findings of the study to each school, principal and its teachers. The researcher will provide this in a format that is most suitable and preferential for each school (e.g. written report, face-to-face meeting, email or telephonic). They will also be given access to the article once the final findings are published.

### Trial status

This is protocol version 5 on 28 June 2016. The protocol was reviewed by the departmental and divisional reviewers at UCT. Following feedback, a rebuttal was submitted. Once the protocol was approved by the departmental and divisional reviewers, the protocol was sent to the FHSREC to obtain ethical approval. The protocol has, therefore, undergone a number of reviews. Recruitment began on the 31 January 2017.

## Additional files

Additional file 1: Figure S2. School stratification and randomisation procedures. **Figure S3.** Graphical representation of the data collection procedure. **Table S1.** Summary of the objectives, outcomes, hypotheses and methods of analysis. (DOCX 49 kb)

Additional file 2: SPIRIT 2013 Checklist: recommended items to address in a clinical trial protocol and related documents. (DOC 122 kb)

## Abbreviations

CCR: Classroom Communication Resource
CONSORT: Consolidated Standards of Reporting Trials
CWS: Children who stutter
FHSREC: Faculty of Health Sciences and Research Ethics Committee
GEE: Generalised estimating equations
ICF: International Classification and Functioning of Disability
ITT: Intention-to-treat
LoLT: Language of learning and teaching
NNSSF: National Norms and Standards for School Funding
PATCS: Peer Attitude Towards Children who Stutter
POSHA-S: Public Opinion Survey of Human Attributes-Stuttering
PRS: Protocol registration and results system
PSD: Positive Social Distance
RCT: Randomised controlled trial
SP: Social Pressure
SPIRIT: Standard Protocol Items for Reporting in Trials
SROM: Stuttering Resource Outcomes Measure
TAB: Teasing and Bullying: Unacceptable Behaviour
UCT: University of Cape Town
VI: Verbal Interaction
