# Supplementary material for: The Classroom Communication Resource (CCR) intervention to change peer’s attitudes towards children who stutter (CWS): study protocol for a randomised controlled trial
Source: Trials. 2018 Jan 17;19:43. doi: 10.1186/s13063-017-2365-x (PMC5773158; doi:10.1186/s13063-017-2365-x)
Supplement: Supplementary file 1 — School stratification and randomisation procedures. Figure S3. Graphical representation of the data collection procedure. Table S1. Summary of the objectives, outcomes, hypotheses and methods of analysis. (DOCX 49 kb) [file 13063_2017_2365_MOESM1_ESM.docx]

**Additional file 1**

**Figure S2: School stratification and randomisation procedures**

Lower quintile schools

Higher quintile schools

Control

Intervention

Intervention

Control

**Figure S3: Graphical representation of the data collection procedure**

| **Timeline** | **Intervention** | **Control** |
| --- | --- | --- |
| **Randomisation** |  | |
| **Baseline (pre-intervention)** | b | |
| **1 week**  **3 week** |  |  |
| **6 months** | **Measure of outcomes** | |

|  | A video of a CWS will be viewed by all participants to ensure that they all have a clear understanding of what stuttering looks and sounds like. |
| --- | --- |
| b | The SROM will be administered to all participants. |
|  | Teacher training will occur, and teachers will be given a two-week period to prepare for the administration of the CCR intervention. |
|  | The teacher will administer the CCR intervention (the story, role-play and discussion). |

**Table S1: Summary of the Objectives, Outcomes, Hypotheses and Methods of Analysis**

| **Objectives** | **Outcome** | **Hypothesis** | **Method of Analysis** |
| --- | --- | --- | --- |
| **Primary:** determine the effect of the CCR vs usual practice (i.e. no CCR) on attitudes around stuttering among grade 7 students at 6-months | Global SROM score | CCR scores will be better than those of usual practice | Generalized estimating equations [GEE] (assuming exchangeable correlation structure within a school) |
| **Secondary:** the effect on attitudes towards stuttering based on SROM subscales | Positive Social Distance (PSD), Verbal Interaction (VI); Social Pressure (SP) | CCR scores will be better than those of usual practice | GEE |
| **Subgroup:** We will also explore subgroup differences between the lower and higher quintile schools. | Global SROM, PSD, VI and SP scores | Effect of CCR vs usual practice will differ by quantile group | GEE with an interaction term of quintile group x intervention |
| **Tertiary:** to conduct a focus group of experts to help with interpreting the trial results. | All outcomes | Not applicable | Qualitative analysis |
